# Views and attitudes of university students in Upper Egypt towards youth health centers

**DOI:** 10.1186/s42506-020-00046-x

**Published:** 2020-09-29

**Authors:** Mohamed M. Abd El-Mawgod, Shimaa A. Elghazally, Heba M. Mohammed, Mariam Roshdy Elkayat, Doaa M. M. Osman

**Affiliations:** 1 Department of Public Health and Community Medicine, Faculty of Medicine, Al-Azhar University, Assiut, Egypt; 2 Occupational and Environmental Medicine, Assiut University, Assiut, Egypt; 3 Public Health and Community Medicine Department, Assiut University, Assiut, Egypt

**Keywords:** Youth, University students, Youth health center, Youth-friendly services

## Abstract

**Background:**

A healthy youth is considered the major human resource for any country development. They are suffering from unmet health needs. Considering these needs and their attitude towards the use of youth health center (YHC) services would help to improve both the quality and quantity of these services.

**Objectives:**

To identify the students’ perceived health needs and their attitude towards use of the YHCs in Assiut University campus, Upper Egypt a cross-sectional study was conducted among 305 randomly selected university students. Data were collected using an interviewer-administered questionnaire.

**Results:**

The majority of the students (80%) said that youth have special health needs. The most reported needs were psychological support, health education on different topics including reproductive health and sexually transmitted diseases, and nutritional services respectively. There was a high perception among surveyed students (71.5%) that the existing health services are inadequate for meeting their needs. Counseling, laboratory services, and premarital examination were the most frequently reported services mentioned by youth to be offered in YHCs. The majority (78.1%) preferred the health provider to be of the same sex. Despite the prevailing conservative culture in Upper Egypt, the students had positive attitude towards availability of sexual and reproductive information and establishment of a YHC in the university campus. A low awareness rate (15.1%) about the already existing YHC in university campus was revealed.

**Conclusion:**

University students perceived that there are unmet needs for youth-specialized services, mainly for providing sexual and reproductive information, and establishment of an on-campus YHC. The study provides important information for policymakers about the perspectives of youth which should be taken into consideration when new YHC are planned and implemented.

## Introduction

Youth is defined as people aged between 15 and 24 years and they are characterized by unique physical, psychological, social, and emotional changes that put their life at high risk [1].

Youth-friendly health centers (YHC) are specialized centers that provide services to young people aged 15–24 in a safe, confidential, and private environment that respects their rights and are attractive to them. Youth centers provide the relevant combination of sexual and reproductive health (SRH) as well as other services such as counseling for puberty changes, and nutrition. Moreover, they offer other preventive, diagnostic, and treatment services [2].

Various factors, such as inaccessibility, inconvenient working hours, unaffordable cost, poor linkages with other relevant services, and lack of youth friendliness, confidentiality, and professional skills, are considered major barriers to proper use of YHCs. In addition, another important barrier in getting access to SRH is the fear of being recognized by parents or people who may be familiar with them. The gender of the health care provider is another influencing factor as young adults could have some problems in explaining SRH issues to a provider of the opposite gender [3–6].

In developing countries, the level of knowledge about the use of youth-friendly services is limited [7]. Among Jordanian youth, for example, knowledge about reproductive health issues was insufficient and they reported their need for reproductive information and qualified services. The main limitations for using SRH services were unsatisfactory facilities and poor professional conduct [8].

In Egypt, several YHCs were established in different areas [9]. Assiut University YHC was established in October 2010**.** It is the first facility that offers reproductive services within the university campus. All elements of success of YHCs were taken into consideration in the planning phase in Assiut YHC such as accessibility, confidentially, design, suitable working hours, competent young health service providers of the same gender for the clients, and promoting YHC through well-trained peers. Moreover, in response to youth interests, the center provides services such as nutrition, mental health, and dermatology consultation. However, Assiut YHC encountered low utilization rate in spite of growing youth needs to knowledge and services. The objectives of this study are to identify the students’ perceived health needs and their attitude towards use of the YHCs as an approach of service delivery in Assiut University campus.

## Participants and methods

### Study design

A descriptive cross-sectional study was conducted in a 6-month duration from March to September 2013 during the academic year 2013–2014. It was carried out as a baseline for continuous monitoring of the use of the services offered by the center.

### Study setting

The study was carried out in Assiut University, Egypt. Total number of faculties at the study time was 15, eight practical, and seven theoretical faculties. In order to have representative sample from both theoretical and practical faculties, four faculties were selected randomly (two theoretical and two practical faculties) namely faculties of Education, Commerce, Medicine, and Veterinary Medicine, respectively***.***

### Participants and sample

The study targeted third- and fourth-year students of both sexes. This group was chosen because they are likely to be preparing for marriage and hence would have more questions and concerns about reproductive health than younger students.

The sample size for the quantitative study was calculated using the following formula for single population proportion:

$$ N=\frac{(z)^2\ P\ \left(1-P\right)\kern0.5em }{d^2}=\frac{(1.96)^2\times 0.1\times (0.9)\ }{(0.05)^2}=138.2976\sim 138 $$

The value of *P* (hypothesized proportion of students having knowledge about YHC) was taken as 10%, *d* = 0.05, and z = 1.96 (*n* = sample size, *p* = proportion, *d* = precision).

Given that the sample was a multistage random one and taking into consideration the design effect and a non-response rate of about 10%, the sample size was doubled to 276.595 and 10% were added so the sample size became 304.25, that is ~ 305.

A total of 305 university students were included. The participants were selected using multistage random sampling. In the first stage, two faculties from the theoretical sciences and two from the applied sciences were chosen randomly. These included the faculties of Education and Commerce, and Medicine and Veterinary Medicine, respectively. In the second stage, after obtaining lists of all sections in each faculty, two sections in each faculty were randomly chosen, one from the third and one from 4th year. From the selected sections, recruitment of students who agreed to participate in the study was done consecutively till completing the required sample from each faculty. Nearly an equal number of students was selected from each chosen faculty.

### Tools of the study

Data collection was conducted by all members of the research team. During the last 30 min of the randomly selected sections, the study aims and informed consent procedures were explained to students. Students who participated in the study responded to a structured interviewer-administered questionnaire, which was completed in about 30–45 min. The reliability of the questionnaire using Cronbach’s alpha for internal consistency was 0.8. The questionnaire included the following sections: personal characteristics, health needs of Assiut University students, students’ opinion and expectations regarding youth-friendly services, and attitudes of students about the Assiut University YHC and its capacity to address their health needs. Students’ attitudes towards reproductive health issues and youth health sites were assessed using six statements. Each response had a four-point Likert scale (ranging from strongly disagree to strongly agree).

### Statistical analysis

The Statistical Package for Social Sciences (SPSS, version 15) [10] was used for data entry and analysis. Descriptive statistics (frequency and percentage) were used to present the distribution of the study population. Chi-squared test was applied for identifying significant difference between categorical variables, and Fisher exact test was applied for expected cells counted less than 5. The significance level was set at 0.05.

## Results

The study sample included 305 university students divided nearly equal across the 4 selected faculties (Commerce, Education, Medicine and Veterinary medicine). Their age ranged from 19 to 23 years, females constituted 56.1% of the sample. Almost all the participants were single (99%). Urban students constituted 52.8% of the sample. Nearly half (49.2%) of the students lived with their families, while nearly one third (32.1%) lived alone in a dormitory and the rest (18.7%) lived alone outside a dormitory.

Table 1 shows that the majority of the students (80%) said that youth have special health needs. When asked to list these special health needs, psychological support was mentioned most often, followed by health education on different topics including reproductive health and sexually transmitted diseases, risks of smoking and addiction, personal hygiene in addition to nutritional services, and follow up care. There was a clear perception that existing health services are inadequate for meeting young people’s needs, where 71.5% disagreed with the statement that existing health services satisfy youth health needs. The cited reasons were as follows, low-quality services (41.3%), absence of services targeting youth (24.3%), and inadequate structure and logistics (hospital numbers, drugs, and equipment) (17.9%) and inefficient providers (17%).

**Table 1:** Students’ perception of youth health needs and their opinions regarding whether the existing services meet those needs

Variable	Frequency (***N*** = 305)	Percent
**If youth have special health needs**		
Yes	244	80.0
No	31	10.2
Don’t know	30	9.8
If yes		
**Listed special health needs of youth (*N*** **= 244)**^**#**^		
Psychological support	95	38.9
Health education	80	32.8
Nutritional services	65	26.6
Follow up care	20	8.2
Others® Fig. 1	13	5.3
**Students’ opinions whether existing health services meet youth health needs**		
Yes	63	20.7
No	218	71.5
Don’t know	24	7.8
**Reasons why existing health services do not meet youth health needs (*N*** **= 218)©**		
Low-quality services	90	41.3
Absence of services targeting youth	53	24.3
Inadequate structure and logistics	39	17.9
Inefficient providers	37	17
Others^**¤**^	11	5

^**#**^More than one answer was allowed

®Others like orthopedic and dermatological problems

**©**More than one answer was allowed among students who reported that existing health services do not meet youth health needs

^**¤**^Others like crowding and long waiting time in private clinics

**Fig. 1:**
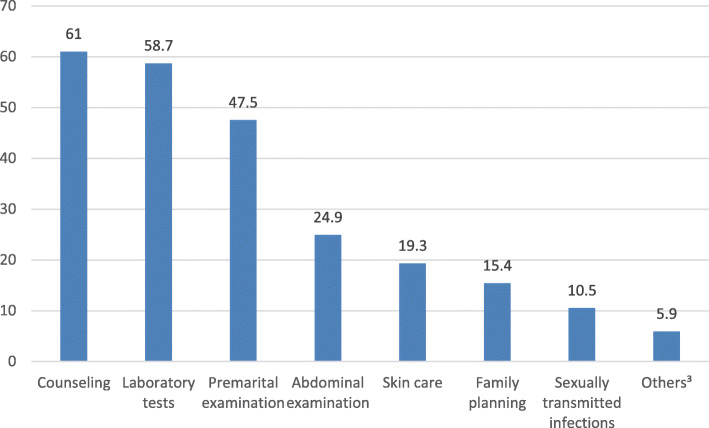
Students’ opinion about health services that should be provided at a youth health facility. More than one answer was allowed. ^¤^Others includes psychological, nutritional care by dietitians, and regular follow up care

Figure 1 illustrates students’ opinions about the specific services that they would like to be offered in a youth health facility; participants were invited to select as many responses as they felt were relevant. The highest percentage of the student (61%) expressed an interest in counseling in general, followed by laboratory tests (58.7%), premarital examinations (47.5%), abdominal examination (24.9%), skin care (19.3%), family planning (15.4%), and management of sexually transmitted infections (10.5%).

Table 2 showed students’ views regarding the characteristics of youth-friendly services. More than 60% of respondents (61.6%) preferred that services be offered on campus, and the most frequently reported reasons were easy accessibility (63.8%), high level of competence (14.9%), and high coverage (13.8%). On the other hand, around one quarter (25.6%) preferred services to be offered off-campus. The most commonly cited reasons were ensuring availability to all youth as a large number of students are outside campus (56.5%), privacy (25.6%), and that the campus is not a suitable place (6.4%). Whether youth services should be offered as stand-alone or be integrated with services for other age groups, there was a clear preference for exclusive youth-friendly services (78.4%), while only 21.6% preferred integrated services. Among the group that preferred stand-alone youth services (*n* = 239), a significant proportion (64%) attributed that to the fact that youth are a vulnerable group with special problems. In contrast, among those who preferred integrated services (*n* = 66), “nearly half (45.5%) said that they preferred integrated facilities to ensure availability of services to everyone, while others (48.5%) believed that there will be no different services targeting youth or discrimination against them”.

**Table 2:** Students’ views regarding youth-friendly services, Assiut University, Egypt

Variable	Frequency (*N* = 305)	Percent
**Site preferred for establishing a youth health facility**		
On campus	188	61.6
Off campus	78	25.6
No preference	39	12.8
**On-campus preference causes (*N*** **= 188)**^**#**^		
Easy accessibility	120	63.8
More efficient	28	14.9
Serve larger number of youth	26	13.8
Low cost of services	8	4.3
Presence of supervision from university authority	7	3.7
Safer	3	1.6
Others	5	2.7
**Off-campus preference causes (*N*** **= 78)**^**#**^		
Availability to all youth	44	56.5
Privacy	20	25.6
Campus not suitable for these services	5	6.4
Better quality of services	5	6.4
Don’t know	4	5.1
**Both on- and off-campus preference causes (*N*** **= 39)**^**#**^		
Serve youth inside and outside campus	34	87.2
No differences	5	12.8
**Students’ opinions about providing youth health services either as stand-alone or integrated**		
Stand alone	239	78.4
Integrated with other services	66	21.6
**Students expectations about health provider at youth health facility**		
Young provider of the same sex	111	36.4
Young provider of either sex	18	5.9
Older provider of the same sex	97	31.8
Older provider of either sex	30	9.8
Age and sex do not matter	49	16.1

^**#**^More than one answer was allowed

Others include; university students should have special privileges and expecting crowdedness if constructed off campus

With regard to the preferred characteristics of health care providers, the majority preferred a provider of the same sex, with slightly more preferring the provider to be young (36.4%) than old (31.8%).

Few questions addressed students’ opinions regarding possible options for the working hours at a youth health facility. Most students preferred that services be offered at non-traditional times, with 41.3% expressing a preference for receiving services in the afternoon or evening, while around a third (32.8%) preferred the weekend. Just 20.3% preferred services during usual working hours.

Table 3 shows the students’ opinions about SRH and, in particular, how they relate to the YHC. The majority of respondents (92.8%) disagreed with the statement that “University students do not need SRH information.” Further, 86.2% indicated that they did not believe that SRH information lead to high-risk sexual behaviors. The majority agreed that SRH educational materials should be available on campus (82%) and that an SRH clinic should be available in campus (88.8%). Meanwhile, only 10.2% agreed that visiting a YHC would indicate that someone was sexually active. Nearly all students (99.3%) did not accept customary marriage.

**Table 3:** Attitudes of the students towards reproductive health issues and youth health sites, Assiut University, Egypt

Variable	Strongly agree	Agree	Disagree	Strongly disagree
	***N*** (%)	***N*** (%)	***N*** (%)	***N*** (%)
**University students do not need SRH information**	1 (0.3)	21 (6.9)	203 (66.6)	80 (26.2)
**RH information lead to high-risk sexual behaviors (*n*** **= 304, male = 133)**^**a**^	10 (3.3)	32 (/10.5)	190 (62.5)	72 (23.7) 6)
**Educational material availability in campus**	47 (15.4)	203 (66.6)	51 (16.7)	4 (1.3)
**It is important to have SRH clinic in campus**	59 (19.3)	212 (69.5)	34 (11.2)	0 (0)
**Using youth health site means engagement in sexual relations**	0 (0)	31 (10.2)	209 (68.5)	65 (21.3)
**Is customary marriage accepted?**	0 (0)	2 (0.7)	21 (6.9)	282 (92.4)

^a^There is one missing case because one student refused to respond for this statement

Table 4 focused on students’ knowledge of the established YHC in the university campus. Only 15.1% of students (46 out of the 305 surveyed) were aware that the YHC existed. Among them, nearly half (52.2%) had learned about the YHC from an advertisement present in prominent areas followed by friends (37%) and health education sessions (6.5%). On asking those who knew about YHC, the location of YHC was known by 63%, and most of them (67.4%) knew services offered at YHC. However, 71.7% did not know the YHC working hours.

**Table 4:** Awareness of the students about the established YHC in Assiut University campus

Variable	Frequency (*N* = 305)	Percent
**Awareness about already established YHC in university campus**		
Yes	46	15.1
No	259	84.9
**Source of awareness of YHC (N = 46)**^**#**^		
Advertisement	24	52.2
Friends	17	37
Used YHC services	5	10.9
Health education sessions	3	6.5
Others	2	4.3
**Students’ utilization of YHC among those who were aware (*N*** **= 46)**		
Yes	5	10.9
No	41	89.1
**Reasons for not using the service (*N*** **= 41)**		
Did not have a health problem	14	34.1
Not enough information about YHC services	8	19.5
Embarrassed to access YHC	7	17.1
Lack of trust of quality of delivered services	5	12.2
Hours not suitable	2	4.9
Price is high	1	2.4
Others	4	9.8

^**#**^More than one answer was allowed

Others include restriction by parents to use these services

Table 5 addresses students’ attitude towards reproductive health issues and some demographic variables. Gender had no significant association with attitude of studied subjects regarding the need of university students to SRH information, the importance of availability of RH educational material and establishing SRH clinic on campus, the concept of using YHC services indicates engagement in sexual relation, and acceptance of customary marriage (*P* value > 0.05). However, males had significantly higher agreement (66.7%) on the notion that RH information might lead to high-risk sexual behaviors compared to females (33.3%) (*P* value = 0.001).

**Table 5:** Distribution of the university student’s attitude towards reproductive health issues and YFC by gender

Variables	Male (***N*** = 134)	Female (***N*** = 171)	***P*** value*
	***N*** (%)	***N*** (%)	
**University students do not need SRH information**			
Strongly agree/agree	8 (36.4%)	14 (63.6%)	0.45
Strongly disagree/disagree	126 (44.5%)	157 (55.5%)	
**RH information would lead to high-risk sexual behaviors (*n*** **= 304, male = 133)**^**a**^			
Strongly agree/agree	28 (66.7%)	14 (33.3%)	0.001
Strongly disagree/disagree	105 (40.1%)	157 (59.9%)	
**RH educational materials availability on campus**			
Strongly agree/agree	113 (45.2%)	137 (54.8%)	0.34
Strongly disagree/disagree	21 (38.2%)	34 (61.8%)	
**It is important to have SRH clinic on campus**			
Strongly agree/agree	116 (42.8%)	155 (57.2%)	0.62
Strongly disagree/disagree	18 (52.9%)	16 (47.1%)	
**Using a YFC means engagement in sexual relations**			
Strongly agree/agree	11 (35.5%)	20 (64.5%)	0.31
Strongly disagree/disagree	123 (44.9%)	151 (55.1%)	
**Is customary marriage accepted?**			
Strongly agree/agree	2 (100.0%)	0 (0.0%)	0.19
Strongly disagree/disagree	132 (43.6%)	171 (56.4%)	

*Chi square test was applied

^a^There is one missing case due to student refused to respond for this statement

## Discussion

Previous studies on YHCs focused on client satisfaction, process of service delivery, and clinic setting. However, limited research investigated the youth perspectives in developing countries [2, 7, 11, 12]. The present study focused on identifying the youth perceived health needs and attitude towards use of the YHCs as an approach of service delivery among Assiut University students.

In this research, the majority (80%) of participants reported that youth have special health needs. Mental health was mentioned most often followed by health education and nutritional services. The participants in Oraby’s study in Egypt (2013) reported the need for SRH services such as information on masturbation and its supposed side effects for young men and hymen and virginity for young women [11]. Khalaf et al. in Jordan found that both male and female youth expressed the need for an awareness related to reproductive health, especially issues related sexual health, maternal issues, and psychological issues [8].

In our study, most of respondents agreed that health services did not meet youth health needs mostly attributing it to low quality while nearly one quarter said that no services for youth were available. Oraby stated that limited space and regulation in some YHCs negatively affect confidentiality and privacy of young people [11]. Similarly, Jordanian youth mentioned low quality of services, inaccessibility, lack of resources, and unqualified health care provider were the problems encountered by them when using reproductive health services [8]. Also, Ghafari et al. in Malaysia found that low professional skills of health care providers mitigated proper use of youth services [3]. DeJong and El-Khoury indicated that government health services generally do not recognize the special needs of young people or foster the environment that supports them [13]. The WHO reported that lacking of adolescent responsive health systems is a problem in much of the world and need to expand coverage and adopt standards in delivering youth services [14]. Also, the UNFPA declared that adolescents’ services in Arabic countries are overlooked by maternal and child services and by services for adults [15].

Regarding participants’ opinions about the specific services that they would like to see offered in a youth-focused clinic, the majority expressed an interest in counseling and laboratory tests and nearly half expressed an interest in premarital examinations. This is consistent with Motuma et al. in their study in Ethiopia who mentioned that reproductive health counseling was utilized by nearly 60% of the study youth participants [7]. Atuyambe et al. in their qualitative study in Uganda indicated that adolescents had multiple needs for reproductive health to be addressed through adolescent-friendly services and in particular young counseling services [16].

Similarly, other studies reported the lack of reproductive knowledge and emphasized the importance of reproductive counseling for young adults. Mohammadi et al. in Tehran, Iran, showed that half of the male adolescents had poor knowledge about reproductive health [17]. Moodi et al. found that premarital couples had poor knowledge on reproductive health [18]. Furthermore, Mosavi et al. in Iran, revealed that the most important problems related to adolescents’ SRH were the lack of accurate information [19]. Khalaf et al. in their qualitative study in Jordan indicated that both male and female youth had a limited reproductive health information [8]. Similarly, Gausman et al. in Jordan reported that Jordanian youth need reproductive health-related information [20].

The study by the FHI-UNFPA reported that young people desire services that respect confidentiality, privacy, and build bridges of trust between them and service providers [12]. Khalaf et al. in Jordan found that youth would like to use youth reproductive health services that are available, attractive, accessible, with proper resources including skilled personnel, and preferably only for youth [8].

Regarding preferences of participants for the characteristics of youth-friendly services, in the current study, students preferred the YHC to be offered on campus. Easy accessibility, delivered with a high level of competence, and serving larger number of youths were the most cited reasons. They preferred the services to be exclusive for youth and explained this preference as youth are a vulnerable group with special problems. FHI-UNFPA reported that YFCs in Egypt should be in places which young people tend to be frequent such as youth centers, sporting clubs, mosques, and churches and provision of the services within an environment that suit their preferences. Furthermore, the need of technically competent and empathetic providers was noted [12].

Most students preferred services to be offered at non-traditional times, while Atuyambe et al. in Uganda showed that adolescents preferred RH services to be available all the time (opening and closing hours) [16].

In the present study, nearly two thirds of respondents preferred the provider to be of the same sex. This agreed with the findings that emphasized preference of youth for providers of the same gender [16, 21]. Lack of health providers of same gender affects negatively the utilization of service in Egypt. Oraby mentioned that absence of a male doctor in some YFCs limited the uptake of services by male clients [11]. The FHI-UNFPA reported that in Egypt doctors of the same sex as their clients would help imparting a feeling of ease between client and physician and contributing to making services youth friendly [22].

The UNFPA reported that a basic right for females and males is to provide them with the tools to understand their sexual responsibilities and enrich their sexual knowledge and awareness [23]. In the present study, the overwhelming majority of respondents agreed that university students need SRH information and realized the importance of availability of SRH educational materials on campus as well as establishing a clinic providing SRH services in campus. In consistence with our results, Motuma et al. in Ethiopia reported that about 70% of the youth respondents reported that the youth should get important information, education, and communication (IEC) on RH at the age of 15 years or older [7].

In the current study, the vast majority indicated that they did not believe that SRH information leads to high-risk sexual behaviors. Similarly, Simbar et al. in Iran found that more than two thirds of Iranian university students did not believe that educating young people about SRH would lead to sexual immorality [24]. A study conducted among male adolescents in Tehran, Iran, concluded that limited adolescents’ knowledge regarding STIs poses a significant threat to their sexual and reproductive health [17].

In the present study, participated students had positive attitude towards SRH either providing information or establishing a clinic in spite of the conservative culture known about the population in Upper Egypt. In the same line, only 10% of participants in this study agreed that visiting a YHC would indicate that someone was sexually active. In contrast, negative attitude was reported in the middle east and north Africa by DeJong et al., where youth reluctance in seeking sexual information was due to fear to be misunderstood as being engaged in sexual relation [25].

Only 15% of students in this study were aware that a YHC existed. In agreement with Senderowitz et al., the lack of awareness and little knowledge of the available SRH services were significant barriers to young clients [26]. Also, Oraby in Egypt revealed that non-beneficiaries of YHCs tended to report that they had simply “never heard about YHCs” [11].

Among the small group who had used the YHC, just over half had heard about it from an advertisement, followed by friends, and health education sessions. In the study conducted in Egypt by Oraby, she found that beneficiaries of YHCs had heard about the clinics through the sessions conducted by peer educators or while accompanying a friend or a relative where governmental YHCs are located [11].

In the present study, students who were aware of the YHC but had never used it reported reasons like they did not have a health problem, did not have enough information about the YHC, and felt shamed about using it. In consistency, Hoggart and Phillips in the UK [27] and Bankole and Malarcher in four countries of Africa (Burkina Faso**,** Ghana, Malawi, and Uganda) recognized that fear of discrimination and disrespect hurdles young people who need to use SRH services [28]. Oraby’s study in Egypt and Mohammadi et al.’s in Iran revealed that all YHCs have under-utilized capacity. They stated that the possible causes of under-utilization of YHCs included, negative attitude of the surrounding community towards youth especially to unmarried youth who culturally are not expected to need SRH services and are stigmatized if they sought care from these services [11, 29].

### Study limitations

Results are not representative to the whole youth in Upper Egypt. They are limited to university students as there are groups of youth who did not join university education. Another limitation is non-use of a standardized questionnaire, studying a limited number of relationships, and the possibility of a desirable response when we use interviews.

## Conclusion and recommendations

There are unmet needs for specialized services for youth, and a high need for providing sexual and reproductive information in addition to establishment of on-campus YHC. Students’ opinions should be considered by stakeholders and health policymakers on planning and implementing YHC. Increasing awareness of students about existing “on campus” YHC is mandatory to improve the utilization rate. It could be attained through different advertisement methods as on the university website, hanged poster in prominent areas in the university and dormitories, and empowerment of the role of peers. Other studies are recommended to explore the attitude and opinion of all youth whether joining university education or not.

## Data Availability

The datasets used and/or analyzed during the current study are available from the corresponding author on reasonable request.

## Abbreviations

YHC: Youth health center
SRH: Sexual and reproductive health
WHO: World Health Organization
SPSS: Statistical Package for Social Science
UNFPA: United Nations Population Fund

## References

[1] WHO. Adolescent health and development: World Health Organization: South-East Asia Region 2019. https://www.who.int/southeastasia/health-topics/adolescent-health.

[2] HPC. Youth-Friendly Reproductive Health Services Policy Brief 2017. Higher Population Council: The Hashemite Kingdom of Jordan; 2017. https://www.share-net-jordan.org.jo/sites/default/files/Youth%20Friendly%20Reproductive%20Health%20Services_0.pdf.

[3] Ghafari M, Shamsuddin K, Amiri M (2014). Barriers to utilization of health services: perception of postsecondary school Malaysian urban youth. Int J Prev Med.

[4] Afifi M (2004). Adolescents’ use of health services in Alexandria, Egypt: association with mental health problems. East Mediterr Health J.

[5] Munthali A, Zakeyo B. Do they match? Adolescents’ realities and needs relating to sexuality and youth friendly service provision in Dowa District, Central Malawi. A report 2011. researchgate.net. https://www.researchgate.net/profile/Bernie_Zakeyo/publication/262881396_Do_They_Match_Adolescents'_Realities_and_Needs_Relating_to_Sexuality_and_Youth_Friendly_Service_Provision_in_Dowa_District_Central_Malawi/links/0a85e53916e04ce8af000000/Do-They-Match-Adolescents-Realities-and-Needs-Relating-to-Sexuality-and-Youth-Friendly-Service-Provision-in-Dowa-District-Central-Malawi.pdf.

[6] Agampodi S, Agampodi T, Piyaseeli U (2008). Adolescents perception of reproductive health care services in Sri Lanka. BMC Health Serv Res.

[7] Motuma A, Syre T, Egata G, Kenay A (2016). Utilization of youth friendly services and associated factors among youth in Harar town, east Ethiopia: a mixed method study. BMC Health Serv Res.

[8] Khalaf I, Moghli FA, Froelicher ES (2010). Youth-friendly reproductive health services in Jordan from the perspective of the youth: a descriptive qualitative study. Scand J Caring Sci.

[9] Oraby D, Soliman C, Elkamhawi S, Hassan R. Assessment of youth friendly clinics in teaching hospitals in Egypt. Fam Heal Int Assess Rep 2008. www.fhi360.org.

[10] SPSS. SPSS Inc (2005). Statistical package for the social sciences (SPSS), version 15.0.

[11] Oraby D (2013). Sexual and reproductive health among young people in Egypt: the role and contribution of youth-friendly services. Sex Educ.

[12] FHI-UNPA (Family Health International- United Nations Population Fund) Meeting adolescent reproductive in Egypt. Final report, 2009. https://www.fhi360.org/sites/default/files/media/documents/Meeting%20Adolescent%20Reproductive%20Health%20Needs%20in%20Egypt%20UNFPA.pdf.

[13] DeJong J, El-Khoury G (2006). Reproductive health of Arab young people. BMJ..

[14] WHO. Adolescent responsive health systems. World Health Organization; 2019. https://www.who.int/maternal_child_adolescent/topics/adolescence/health_services/en/.

[15] UNFPA (United Nations Population Fund). Sexual and reproductive health: a core component of universal health coverage. New York: UNFPA Arab States Regional Office position paper; 2014. Available from https://arabstates.unfpa.org /en/ publications / position-paper-sexual-and-reproductive-health-arab-states. Accessed 13 Aug 2019.

[16] Atuyambe L, Kibira P, Bukenya J, Muhumuza C, Apolot RR, Mulogo E (2015). Understanding sexual and reproductive health needs of adolescents: evidence from a formative evaluation in Wakiso district, Uganda. Reprod Health.

[17] Mohammadi M, Farideh K, Zare S, Tehrani F, Ramezankhani A, Alaeddini A (2006). Reproductive kowledge, atitudes and behavior among adolescent males in Tehran, Iran. Int Fam Plan Perspect.

[18] Moodi M, Miri M, Sharifirad GR. The effect of instruction on knowledge and attitude of couples attending pre-marriage counseling classes. J Educ Health Promot. 2013;2(52). 10.4103/2277-9531.119038 PMC3826018 24251288

[19] Mosavi SA, Babazadeh R, Najmabadi KM, Shariati M (2014). Assessing Iranian adolescent girls’ needs for sexual and reproductive health information. J Adolesc Health.

[20] Gausman J, Othman A, Hamad IL, Dabobe M, Daas I, Langer A (2019). How do Jordanian and Syrian youth living in Jordan envision their sexual and reproductive health needs? A concept mapping study protocol. BMJ..

[21] Braeken D, Rondinelli I (2012). Sexual and reproductive health needs of young people: Matching needs with systems. Int J Gynaecol Obstet.

[22] FHI-UNFPA (Family Health International- United Nations Population Fund) 2008. Assessment of Youth Friendly Clinics in Teaching Hospitals in Egypt. https://www.fhi360.org/sites/default/files/media/documents/Assessment%20of%20Youth%25.

[23] UNFPA (United Nations Population Fund). Sexual and reproductive health for all: Reducing poverty, advancing development and protecting human rights United Nations. UNFPA; 2010. http://www.unfpa.org/publications/.

[24] Simbar M, Ramezani F, Hashem Z (2003). The needs of reproductive health needs of university students (in Persian). J Qazvin Univ Med Sci.

[25] DeJong J, Shepard B, Roudi-Fahimi F, Ashford L. Young people’s sexual and reproductive health in the middle east and north Africa. PRB (Population Reference Bureau) Young People’s Sexual and Reproductive Health in MENA; 2007. https://www.prb.org/menayouthreproductivehealth/.

[26] Senderowitz J, Hainsworth G, Solter C. Rapid assessment of youth friendly reproductive health services. Pathfinder Interrnational. 2003;4. https://www.pathfinder.org/publications/rapid-assessment-youth-friendly-reproductive-health-.

[27] Hoggart L, Phillips J (2011). Teenage pregnancies that end in abortion: what can they tell us about contraceptive risk taking?. J Fam Plann Reprod Health Care.

[28] Bankole A, Malarcher S (2010). Removing barriers to adolescents’ access to contraceptive information and services. Stud Fam Plann.

[29] Mohammadi F, Kohan S, Mostafavi F, Gholami A (2016). The Stigma of reproductive health services utilization by unmarried women. Iran Red Crescent Med J.

